# Molecular Targets for Combined Therapeutic Strategies to Limit Glioblastoma Cell Migration and Invasion

**DOI:** 10.3389/fphar.2020.00358

**Published:** 2020-03-27

**Authors:** Andrea J. Yool, Sunita Ramesh

**Affiliations:** ^1^Adelaide Medical School, University of Adelaide, Adelaide, SA, Australia; ^2^College of Science and Engineering, Flinders University, Adelaide, SA, Australia

**Keywords:** ionotropic glutamate receptor, synaptic protein expression, transcriptomic, ion channels, traditional herbal medicines, aquaporin, glioblastoma

## Abstract

The highly invasive nature of glioblastoma imposes poor prospects for patient survival. Molecular evidence indicates glioblastoma cells undergo an intriguing expansion of phenotypic properties to include neuron-like signaling using excitable membrane ion channels and synaptic proteins, augmenting survival and motility. Neurotransmitter receptors, membrane signaling, excitatory receptors, and Ca^2+^ responses are important candidates for the design of customized treatments for cancers within the heterogeneous central nervous system. Relatively few published studies of glioblastoma multiforme (GBM) have evaluated pharmacological agents targeted to signaling pathways in limiting cancer cell motility. Transcriptomic analyses here identified classes of ion channels, ionotropic receptors, and synaptic proteins that are enriched in human glioblastoma biopsy samples. The pattern of GBM-enriched gene expression points to a major role for glutamate signaling. However, the predominant role of AMPA receptors in fast excitatory signaling throughout the central nervous system raises a challenge on how to target inhibitors selectively to cancer cells while maintaining tolerability. This review critically evaluates a panel of ligand- and voltage-gated ion channels and synaptic proteins upregulated in GBM, and the evidence for their potential roles in the pathological disease progress. Evidence suggests combinations of therapies could be more effective than single agents alone. Natural plant products used in traditional medicines for the treatment of glioblastoma contain flavonoids, terpenoids, polyphenols, epigallocatechin gallate, quinones, and saponins, which might serendipitously include agents that modulate some classes of signaling compounds highlighted in this review. New therapeutic strategies are likely to exploit evidence-based combinations of selected agents, each at a low dose, to create new cancer cell-specific therapeutics.

## Glioblastoma Invasiveness Severely Limits Patient Survival

The prospects for survival of patients diagnosed with glioblastoma are poor, even with the most powerful therapies, largely because of the highly invasive nature of the cancer. After diagnosis, average survival expectancy ranges from a few months to less than 2 years, depending on cancer subtype and treatment strategy (Stupp et al., 2005). As reviewed recently, glioblastoma is divided into primary GBM, constituting 80% of cases with a mean age of onset at 62 years, and secondary GBM progressing from lower grade astrocytomas or oligodendrogliomas with a mean age of onset of 45 years (Ghosh et al., 2018). Glioblastoma multiforme is designated as Grade IV by the World Health Organization, the most malignant type of brain tumor. Molecular markers that have been defined for GBM include DNA repair and methylation enzymes, epidermal growth factor receptor, proto-oncogenes, and tumor suppressor genes (Ghosh et al., 2018). Current treatments for GBM depend on a combination of surgical resection, radiation therapy and chemotherapy, but even with this multi-pronged approach, median survival time is only 14.6 months (Ghosh et al., 2018).

In historical records, the first intracranial procedure in 1884 to remove a glial tumor was attributed to Bennett and Godlee (Walker et al., 1976). Surgical resection can remove most of a tumor bulk, but the problem is that a subset of GBM cells at the time of treatment will have already spread a substantial distance beyond the visible boundary of the main mass. Postmortem analyses of sectioned whole brains from untreated GBM patients revealed that in none of the 15 cases studied did the computer-tomography defined area capture the full extent of the neoplasm, and in three cases, infiltration extended 3.5 to 5 cm beyond the observed electron-dense boundaries of the tumors (Burger et al., 1988). Conventional radiological evaluations frequently underestimate the full area over which an invasive glioma has extended (Claes et al., 2007). In the late 1970s, addition of radiotherapy after surgery was shown to further improve GBM patient survival, and has since been refined progressively to optimize focused applications (Mann et al., 2017). Radiation damage to DNA acts to decrease cell proliferation, but falls short of being curative as a result of the invasive nature of GBM, as well as radiation-induced necrosis, neuronal damage, and the insensitivity of some GBM subtypes to radiation treatment. Adjuvant chemotherapy was found to further improve survival. In 2005, a Phase 3 clinical trial conducted by the European Organization for Research and Treatment of Cancer (EORTC) 26981-22981/National Cancer Institute of Canada Clinical Trials Group (NCIC CTG) showed significantly better overall survival when patients were treated with temozolomide in combination with radiotherapy and maximal surgical resection (Stupp et al., 2005). However, recurrence remains a serious problem, as GBM cells escape resection by early migration, and show inherent or acquired resistance to chemo- and radio therapy treatments (Ghosh et al., 2018). More work is needed to understand the mechanisms of motility, to define new intervention strategies for this devastating disease, and potentially to aim treatments to specific subtypes of GBM.

GBM arises throughout the brain most frequently in the cerebral hemispheres, particularly in frontal lobes or both frontal and temporal lobes, whereas it is relatively rare in cerebellum (Iacob and Dinca, 2009). Categories of glioblastoma traditionally were defined based on histology; recently a systematic correlation of phenotypes with molecular markers identified diagnostic indicators of the major subtypes (Louis et al., 2016; Verhaak, 2016). The molecular markers themselves are not necessarily key drivers of glioblastoma pathology, but their usefulness in defining subtypes is expected to allow tailoring of treatment aggressiveness to optimize patient outcomes (Verhaak, 2016). Frequently altered pathways in cancers involve tyrosine and serine/threonine kinases and relevant receptors, including the epidermal growth factor receptor (EGFR), which alter the regulation of Ras oncogenes overexpressed in glioblastoma (Cancer Genome Atlas Research, 2008). Downstream cascades of kinases include the mitogen-activated protein kinase family that are associated with an array of outcomes including proliferation, invasion, and chemotherapy resistance. We suggest that additional contributions from neurotransmitters, membrane signaling, excitatory receptors, and Ca^2+^ responses could be similarly important, and might offer opportunities for customized treatment of cancer cells within the heterogeneous cell populations of the central nervous system by combinations of agents at subtoxic doses.

Limiting cell migration is a compelling goal in the broad cancer field. Tools for managing the invasive spread of cancer cells in general through the body are essential, given that metastasis is a major cause of cancer-related deaths (Welch and Hurst, 2019). In GBM, the cancer rarely metastasizes outside the central nervous system, yet within the brain the highly invasive properties of these cells constitute a major challenge for effective treatment. Controlling GBM spread using inhibitors of cell migration and invasion would constitute a powerful adjunct therapy that could be applied in parallel with other procedures aimed at direct eradication of primary tumors. This review critically evaluates classes of ligand- and voltage ion channels and synaptic proteins as new targets for experimental therapies in GBM, and integrates cross-disciplinary evidence for their potential roles in the pathological features of glioblastoma.

## Pharmacological Targets for Cancer Treatment

**Figure 1:**
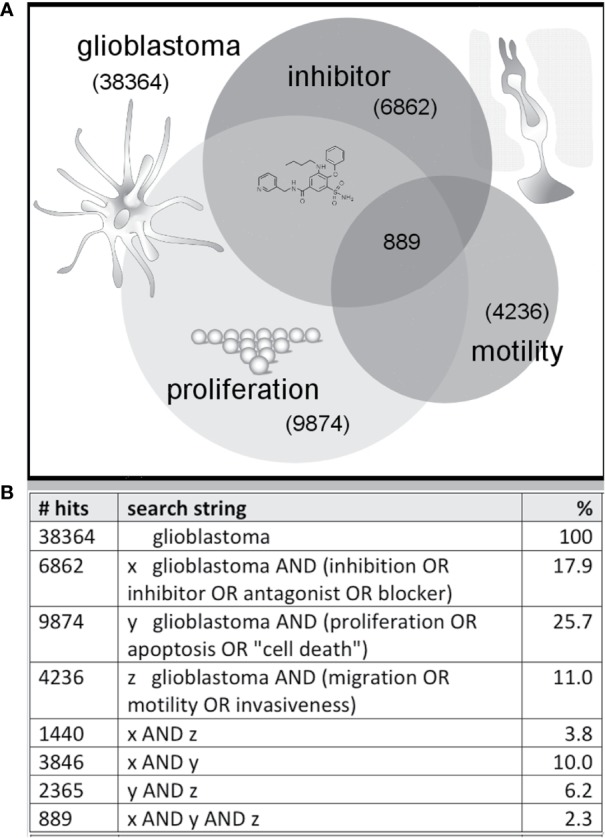
Illustration of a sample distribution of published glioblastoma studies suggesting less than 3% combine three themes (inhibitor, proliferation, motility). Venn diagram **(A)** summarizing the numbers of published articles on glioblastoma, with key words linked to inhibition, migration and growth, as of November 2019 based on a search of the NIH PubMed database, with search strings as defined in the table **(B)**.

The majority of published work in the drug discovery field for cancers including GBM has focused on cell death and reduced proliferation as the primary outcomes of interest for therapeutics. While these candidate agents would have desired effects of killing cancer cells, non-selective toxicity, side-effects, and efficacy *in vivo* remain constraints on effective drug development. Alkylating agents that modify DNA structure have been shown to improve patient survival by driving apoptosis of the cancer cells, but concurrent activity on non-cancerous cells creates side effects which limit tolerable doses. Alkylating agents for GBM include chloroethylating drugs such as carmustine and lomustine, and methylating agents such as temozolomide which, due to its comparatively lower toxicity, has in combination with radiotherapy become a standard of care for GBM patients in countries that can afford the high cost of this chemotherapeutic agent (Lonardi et al., 2005). A second tier of cytotoxic agents for non-responsive GBM cases includes carboplatin, etoposide, oxaliplatin, and irinotecan. These agents also alter DNA to reduce cell proliferation, with greatest effects exerted on populations of rapidly dividing cells such as cancers. Anti-angiogenic agents and antibodies against EGFR and other tyrosine kinase receptors also have been of interest for new experimental chemotherapy strategies (Iacob and Dinca, 2009). A major gap in knowledge in this field is how to constrain GBM cell motility while treatments of the primary tumor masses are in progress, preventing the escape that leads to recurrence. As summarized in this review, the discovery of pharmacological tools to intervene in processes of cell migration and invasion in GBM is a promising area of work, possibly utilizing traditional medicinal herbs as one source of novel agents, but this area remains largely unexplored to date. As an indicative survey, of more than 38,000 papers listed in the National Institutes of Health PubMed database that were identified as relevant to glioblastoma as of Nov 2019, 18% identified use of an inhibitor, 26% were linked to proliferation, and 11% considered effects on motility (**Figure 1**). Less than 4% of published studies in glioblastoma evaluated candidate therapeutics as tools for limiting cancer cell motility. Approximately 2% of the papers published evaluated the effects of inhibitors on both cell survival and motility.

Evidence suggests combinations of therapies could be more effective than single agents alone. For example, a Chinese traditional medicinal extract known as Compound Kushing Injection has been fractionated, chemically defined, and evaluated for dose-dependent activities in a broad array of cancer cell types measured by changes in rates of migration, cell survival, proliferation, and gene expression (Nourmohammadi et al., 2019). The interesting conclusions drawn in this work were that no single compound in the Compound Kushing Injection mixture accounted for the full therapeutic effect of the whole extract; combinations of agents were required to reconstitute the beneficial activity, and the most effective combinations of compounds depended on the cancer cell type. Combined block of glioma cells with 5-nitro-2-(3-phenylpropylamino)-benzoate (a non-specific chloride channel blocker), covalently linked to temozolomide (an oral chemotherapeutic drug for glioblastoma) suppressed glioma cell proliferation, migration, and invasion (Park et al., 2016). Temozolomide modifies DNA or RNA at guanine and adenine bases by addition of methyl groups (Lee, 2016); however, a substantial proportion of tumors are insensitive to temozolamide due to expression of methyltransferase genes.

Limitations of current therapies highlight the need for new drugs that induce fewer side effects and can lead to a more favorable prognosis. While the combination of radiotherapy and temozolomide is a principal treatment strategy, a rising concern is that radiation exposure at therapeutic levels can alter gene expression and signaling to promote a highly invasive phenotype in the surviving glioblastoma cells (Birch et al., 2018). Promising results have indicated that a blocker of a key kinase (which regulates cytoskeletal contractility at the leading edges of glioblastoma tumors) could be co-administered to suppress the recurrence of the invasive cells after radiotherapy, with potential promise for extending patient survival time and reducing the neurological impairment associated with the disease (Birch et al., 2018). A major active ingredient of the herbal medicinal plant ginseng (ginsenoside Rg3), has additive anti-tumor and anti-angiogenic effects when applied in combination with temozolomide (Sun et al., 2016b). Epigallocatechin gallate from green tea synergizes with temozolomide to inhibit viability, migration, and drug resistance of glioma stem-like cells isolated from the U87 line (Zhang et al., 2015). The anti-invasive activity of a medicinal herbal extract from olive (*Olea*) leaf on glioblastoma stem cells derived from human primary tumors was increased when given in combination with bevacuazimab (Tezcan et al., 2017a). Further work seems likely to demonstrate that the most effective clinical strategies will include co-administration of multiple agents, targeted to subtype-specific panels of factors in glioblastoma, to enhance the successful outcomes of interventions.

## Mechanisms Enabling Glioblastomal Progression

High motility, heterogeneity of cell types within the cancer clusters, as well as the ability of the glioblastoma cells to transition between proliferative and non-proliferative phases make these cancers difficult to treat, and complicate the accurate diagnoses of tumor types based on small biopsies (Scherer, 1940; Singh et al., 2004; Behnan et al., 2019). Migration pathways of glioblastoma through the human brain, described by Scherer (1940) and confirmed in rodents with human tumor cell implants (Laws et al., 1993), suggest a predominant pattern of reliance on myelinated tracts such as corpus callosum, basement membranes of blood vessels, pial surfaces, and subependymal layers surrounding the ventricles as routes for invasion. Insights from Scherer's work further indicated that interactions with the brain tissue environment were an important part of glioblastoma progression, a concept highly relevant to current discoveries.

Surprising capacities for membrane signaling in glioblastoma are reminiscent of properties characteristic of excitable cells, and appear to be important for disease progression (Soroceanu et al., 1999; Catacuzzeno et al., 2011; Molenaar, 2011; Wong et al., 2017; Venkataramani et al., 2019; Venkatesh et al., 2019). Although glioblastoma cells have been thought to arise from glia in the CNS, molecular evidence indicates they originate from stem cell precursors (Emlet et al., 2014), and undergo an intriguing expansion of phenotypic properties to include neuron-like signaling, which has been proposed to augment glioblastoma cell survival and motility (Venkataramani et al., 2019). As analyzed here, a select set of excitable membrane ion channels and synaptic proteins (usually assumed to be the province of cells such as neurons, not glia) are upregulated in glioblastoma. This unusual portfolio of signaling proteins could endow glioblastoma cells with functional capabilities that aid their robust persistence and merit investigation as targets for new therapeutics, though the signaling protein contributions to the underlying pathological mechanisms are yet to be fully defined.

Glutamate, the main excitatory neurotransmitter in the central nervous system, in early brain development promotes the proliferation of neuronal progenitor cells, but interestingly also has been found to enhance proliferation and migration in glioblastoma cells (Lyons et al., 2007; Corsi et al., 2019). Glutamate responses associated with glioblastoma are more than excitotoxicity mechanisms to kill neurons and create space for tumor growth (Sontheimer, 2008); the process is an integral part of bidirectional communication between neurons and glioblastoma cell networks (Robert and Sontheimer, 2014; Ribeiro et al., 2017; Corsi et al., 2019). Excitation of AMPA-activated classes of ionotropic glutamate receptors (GluA1 to 4) augments the cell migration and proliferation of human glioblastoma cells (Ishiuchi et al., 2002), mediated in part by Ca^2+^-dependent activation of a survival factor Akt kinase, as confirmed in surgical samples and in glioblastoma cell lines (Ishiuchi et al., 2007). Pathological tumor cells are not unique among non-neuronal brain cells in being responsive to glutamate; immature glia also show sensitivity. In oligodendrocyte precursor cells, functional synapses marked by fast depolarizing currents were measured in response to voltage- and calcium-dependent release of glutamate from pyramidal neurons in hippocampal slice preparations (Bergles et al., 2000). During oligodendrocyte maturation, signaling from axons is proposed to coordinate the process of myelination. In human glioblastoma, synapses from neurons onto glioblastoma cell processes were observed by electron microscopy in resected tumor samples, complete with hallmark structures including presynaptic docked vesicles, synaptic clefts, and postsynaptic densities carrying AMPA receptors, confirmed by molecular markers and electrophysiological recordings (Venkataramani et al., 2019). Prior work had showed glutamate-induced Ca^2+^ signals and suggested the occurrence of action potentials in some dissociated cells cultured from glioma samples (Labrakakis et al., 1998), though cell types in the mixed sample were not precisely identified. Postsynaptic responses characterized in identified glioblastoma depend directly on the activity of surrounding neurons and seem focused on increasing intracellular Ca^2+^; no action potentials were observed in more than 300 tumor cells recorded (Venkataramani et al., 2019). Signals driven by synaptic input spread through the gap-junction linked network of glioma cells to promote proliferation, and reciprocally drive hyperexcitability of neurons in the glioma microenvironment, further boosting glioblastoma synaptic input and amplifying the problem (Venkatesh et al., 2019). We propose that analysis of a transcriptomic database with a focus on classes of ion channels, ionotropic receptors, and synaptic proteins that are enriched in human glioblastoma biopsy samples, offers a timely opportunity to generate predictive hypotheses for further testing the possible functional roles of these signaling proteins in glioblastoma progression.

Quantitative data on transcript levels measured from biopsied samples from human glioblastoma multiforme (GBM) patients, available in the Human Protein Atlas, were queried here to compare the patterns of gene expression of an array of excitable membrane signaling proteins for GBM patients with the likelihood of their survival of at 3 years after diagnosis (Uhlen et al., 2005; Uhlen et al., 2017). **Table 1** provides a summary of compiled data for the classes of ionotropic receptors, ion channels, and synaptic proteins that showed distinct upregulation in measured transcript levels in GBM patients. Transcriptomic data were based on analyses of biopsied samples from 153 patients diagnosed with GBM (54 female and 99 male). At the time of the data collection, 123 patients were deceased. Transcript levels were quantified as ‘Fragments per kilobase of transcript per million fragments mapped' (FPKM). The percent survival at 3 years is calculated from deceased patients whose samples showed transcript levels for a given signaling protein greater than the median value across the GBM group; these values ranged from 3% to 14%, depending on the gene. Several genes were significant as negative prognostic indicators of survival when upregulated, including the small-conductance Ca^2+^-activated K channel (KCa3.1), synaptotagmin 5, gap junction channel connexin 26, and the Na^+^ proton exchanger NHE1 (though upregulation of this transporter was seen in multiple types of cancers, not selectively in GBM). Among the classes of ionotropic glutamate receptors enriched in GBM patients (**Table 1**), transcript levels were high for the AMPA-activated classes GluA1, GluA2, and GluA3, and the kainate-activated class GluK5. Ionotropic receptors for GABA and acetylcholine were enriched in GBM, but were not expressed at levels as high as those seen for glutamate receptors. Analyses of gene expression for the voltage-gated ion channel family showed expression of Ca_V_ and Na_V_ channels, as well as multiple types of K^+^ channels. The highest levels of ion channel transcripts were seen for the inward rectifier K^+^ channel Kir4.1, and were appreciable as well for voltage-gated Kv4.2 and Kv7.2, and Ca^2+^-activated K_Ca_2.3 channel classes. High levels of synaptic proteins were seen for synaptosome-associated protein SNAP25, synaptic vesicle glycoprotein, synapsin, synaptophysin, and synaptotagmins. Other channels with higher levels of expression in GBM include Aquaporins 1 and 4, the acid-sensing channel ASIC1, cyclic nucleotide gated channel CNG3, and the chloride channel ClC3. Though downregulation of expression of other genes in GBM also could contribute to pathological features of the disease, work here has focused on the genes showing upregulation of transcript levels, based on the proposed goal of finding potential therapeutic targets for novel inhibitory agents.

**Table 1:** Summary of ion channels and synaptic proteins showing enriched transcript levels (FPKM, fragments per kilobase of transcript per million fragments mapped) in human glioblastoma biopsy samples, analyzed using TGCA RNAseq (n=153; Human Protein Atlas database); and percent survival at 3 years for patients with FPKM values greater than median.

Gene	Classification	Patholog evidence	FPKM, median	% survival
**Glutamate ionotropic receptors**				
GRIA1 (GluA1, GLUR1, GLURA)	ionotropic AMPA type 1	1	7.5	7
GRIA2 (GluA2, GLUR2, GLURB)	ionotropic AMPA type 2	1	5.6	11
GRIA3 (GluA3, GLUR3, GLURC, MRX94)	ionotropic AMPA type 3	1	8.5	10
GRID2 (GluD2, GluR-delta-2)	ionotropic delta type 2	1	0.9	13
GRIK4 (GluK4, GRIK, KA1)	ionotropic kainate type 4	1	3.05	13
>GRIA4 (GluA4, GLUR4, GLURD)	ionotropic AMPA type 4	0	4.7	14
GRID1 (GluD1, KIAA1220)	ionotropic delta type 1	0	3.4	7
GRIK1 (GluK1, GLUR5)	ionotropic kainate type 1	0.25	3.2	7
GRIK2 (GluK2, GLUR6, MRT6)	ionotropic kainate type 2	0.5	2.8	13
GRIK3 (GluK3, GLUR7)	ionotropic kainate type 3	0	11	9
GRIK5 (GluK5, GRIK2, KA2)	ionotropic kainate type 5	0	14.9	14
**Gamma-aminobutyric acid (GABA) receptors**				
GABRA1 (EJM5)	GABA- A alpha1	1	0.3	12
GABRG1	GABA-A gamma1	0.5	0.2	11
GABRB1	GABA-A beta1	0.25	0.5	5
GABRA2	GABA-A alpha2	0	0.4	6
GABRG2	GABA-A gamma2	0	0.4	11
**Nicotinic cholinergic receptors**				
CHRNA1 (CHRNA)	Cholinergic nicotinic alpha 1	1	0.9	6
CHRNA9 (NACHRA9)	Cholinergic nicotinic alpha 9	0	1.4	12
CHRNB2	Cholinergic nicotinic beta 2	0	1.3	3
**Calcium voltage-gated channels**				
CACNG3	voltage-gated Ca^2+^ channel gamma 3, brain	1	0.2	12
CACNG8	voltage-gated Ca^2+^ channel gamma 8, brain	1	1.1	8
CACNG5	voltage-gated Ca^2+^ channel gamma 5	0.25	0.1	6
CACNG7	voltage-gated Ca^2+^ channel gamma 7	0	27.9	12
**Potassium voltage-gated and calcium-activated channels**				
KCNA2 (HK4, Kv1.2)	voltage-gated K^+^ channel subfamily A 2, brain	1	1.0	14
KCNC1 (Kv3.1)	voltage-gated K^+^ channel subfamily C, brain	1	0.9	5
KCND2 (KIAA1044, Kv4.2, RK5)	voltage-gated K^+^ channel subfamily D 2, brain	1	4.3	12
KCNJ10 (Kir1.2, Kir4.1)	voltage-gated K^+^ channel subfamily J 10, high in brain, moderate in kidney	1	22.8	10
KCNJ9 (GIRK3, Kir3.3)	Gprot activ inward rectifier K^+^ channel subfamily J 9, brain	1	1.7	7
KCNN3 (hSK3, KCa2.3, SKCA3)	Ca^2+^-activated K^+^ channel subfamily N 3, brain	1	4.3	8
KCNQ2 (BFNC, EBN, EBN1, ENB1, HNSPC, KCNA11, Kv7.2)	voltage-gated K^+^ channel subfamily Q 2, M channel, brain	1	7.2	7
KCNN2 (hSK2, KCa2.2)	Ca^2+^-activated K^+^ channel subfamily N 2	0.25	2.6	11
KCNJ4 (HIR, hIRK2, HRK1, IRK3, Kir2.3)	voltage-gated K^+^ channel subfamily J 4	0	1.3	12
KCNN4 (hIKCa1, hKCa4, hSK4, IK, KCa3.1)	Ca^2+^-activated K^+^ channel subfamily N 4 *(not GBM enriched)*	0.5	0.9	8 *
**Sodium voltage-gated channels**				
SCN2A (HBSCI, HBSCII, Nav1.2, SCN2A1, SCN2A2)	voltage-gated Na^+^ channel alpha 2, brain	1	1.1	7
SCN1A (FEB3, GEFSP2, HBSCI, NAC1, Nav1.1, SCN1, SMEI)	voltage-gated Na^+^ channel alpha 1	0	1.6	13
SCN3A (Nav1.3)	voltage-gated Na^+^ channel alpha 3	0.5	2.3	11
SCN3B (HSA243396)	voltage-gated Na^+^ channel beta 3	0	1.3	6
**Synaptic proteins**				
NSG2 (CALY3, HMP19, Nsg2)	Neuronal vesicle trafficking associated 2	1	4.0	10
SNAP25 (bA416N4.2, dJ1068F16.2, RIC-4, RIC4, SEC9, SNAP, SNAP-25)	Synaptosome associated protein 25	1	14.4	12
SNAP91 (AP180, CALM, KIAA0656)	Synaptosome associated protein 91	1	1.3	9
SV2A (KIAA0736, SV2)	Synaptic vesicle glycoprotein 2A	1	26.5	7
SYN1	Synapsin I	1	5.9	9
SYNPR (MGC26651, SPO)	Synaptoporin	1	0.4	9
SYP (MRX96)	Synaptophysin	1	10.3	6
SYT5	Synaptotagmin 5	1	1.2	11 *
SYT11 (DKFZp781D015, KIAA0080, MGC10881, MGC17226)	Synaptotagmin 11	1	115	10
STX1B (STX1B1, STX1B2)	Syntaxin 1B	0.75	3.9	10
SYN2 (SYNII, SYNIIa, SYNIIb)	Synapsin II	0	2.3	13
SYT6	Synaptotagmin 6	0	1.4	11
**Other channels and transporters**				
AQP1	Aquaporin 1	1	240	12
AQP4 (MIWC)	Aquaporin 4	1	180	9
BEST1	Bestrophin 3 Ca^2+^-activated chloride channel	1	1.7	9
ASIC1 (ACCN2, BNaC2, hBNaC2)	Acid sensing ion channel subunit 1	0	10.4	7
CNGA3 (ACHM2, CCNC1, CCNCa, CNCG3, CNG3)	Cyclic nucleotide gated channel alpha 3	0.5	5.3	8
GJB2 (Cx26)	Gap junction protein beta 2	0.25	2.2	5 *
HCN2 (BCNG-2, BCNG2, HAC-1)	Hyperpolarization activated cyclic nucleotide gated K^+^ and Na+ channel 2	0	6.3	10
SLC9A1 (APNH, NHE1, PPP1R143)	Solute carrier family 9 member A1 *(not GBM enriched)*	1	6.5	6 *
CLCN3 (ClC3)	Chloride voltage-gated channel *(not GBM enriched)*	0.5	19.0	13

(*) indicates a confirmed unfavorable prognostic indicator in glioblastoma. Highlighted rows (yellow) indicate high scores for pathological evidence (1/1), supported by RNA, protein and histological data.

## Transcriptomic Analyses of Ion Channel Genes Selectively Enriched in Glioblastoma

**Figure 2:**
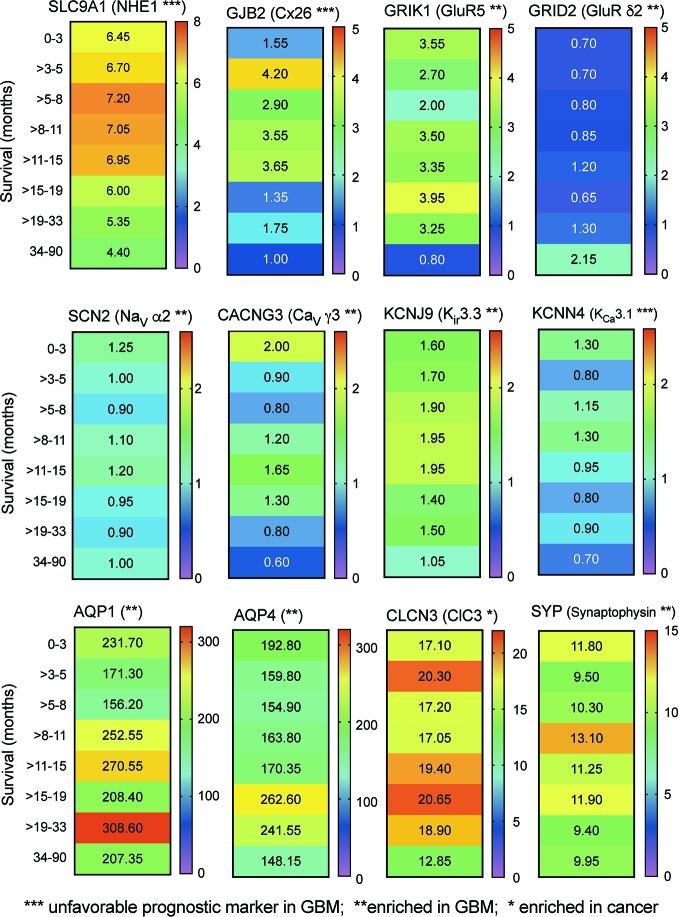
Correlation between relative expression levels of key genes and patient survival. Heat maps summarizing the quantitative median levels of transcripts (FPMK, color bars) for 12 genes, for results banded into bins based on durations of patient survival. Results were calculated from TCGA data available in the Human Protein Atlas. Asterisks indicate relative prognostic values: *** increased transcript expression is an unfavorable prognostic marker in GBM; ** transcript levels are selectively enriched in GBM; * transcript levels are enhanced in cancers.

Transcriptomic data from RNAseq analysis of human glioblastoma samples (Human Protein Database) were further analyzed by banding data into pooled subsets based on patient survival duration, for intervals ranging from 0–3 months up to more than 3 years, and calculating the transcript levels (median FPKM values), depicted as a heat map (**Figure 2**). The negative prognostic indicators NHE1 and Cx26 as expected showed a general pattern in which GBM patients with the highest levels of transcript expression showed the shortest average survival times. A similar pattern might be suggested by data for the voltage gated Ca^2+^ channel, inward rectifier K^+^ channels, and Ca^2+^-activated K^+^ channel classes shown. No distinct correlation between survival and transcript level was evident in this analysis for AQP1 and ClC3 channels, both identified previously as targets of interest in pathological mechanisms of glioblastoma progression (McCoy and Sontheimer, 2007; Cuddapah and Sontheimer, 2010; El Hindy et al., 2013). However, this link between expression and survival is based on small biopsies of heterogeneous tumor masses, and does not rule out potential impacts for any of the channels or synaptic proteins that are enriched in GBM. Signaling molecules often have overlapping functions; thus, analyses that consider sets of related channels within a class, rather than a specific individual channel, might potentially be more meaningful predictors of survival outcomes (see further discussion and **Figure 4** below).

## Glutamate Receptors Enriched in Glioblastoma

Neurons in the central nervous system express diverse classes of direct ligand-gated ion channels that are activated by changes in membrane voltage, by the binding of intracellular ligands such as Ca^2+^ and cyclic nucleotides, by the binding of extracellular ligands such as neurotransmitters, or a combination. Glioblastoma cells express a subset of receptors and channels which are associated with excitable cells, and are of substantial interest for their potential roles in signaling pathways that promote the aggressive and lethal properties of the glioblastoma tumor. Of the direct ligand gated channels, the highest transcript levels were for glutamate receptor genes, with comparatively low levels seen for GABA_A_ and nicotinic acetylcholine receptors (**Table 1**).

Glutamate is the major excitatory neurotransmitter in the brain and spinal cord, released from presynaptic terminals in response to an increase in intracellular Ca^2+^ mediated through voltage-gated Ca^2+^ channels, and detected at postsynaptic glutamate receptors, resulting in an excitatory change in membrane potential. As reviewed by Traynelis and colleagues (2010), direct ligand-gated glutamate receptors are cation-permeable channels built as tetramers of subunits, and have been subdivided by selective agonist sensitivities into the N-methyl-D-aspartate (NMDA) and the non-NMDA classes (AMPA, alpha-amino-3-hydroxy-5-methyl-4-isoxazolepropionic acid, and kainate), categories which align with genetic classifications based on amino acid sequence homology. Transcripts enriched in glioblastoma RNA seq data include receptors in the non-NMDA group activated by the agonist AMPA (GluA1 to -4) and by kainate (GluK1 to -5).

A key determinant of ionic permeability of AMPA and kainate receptors is the type of amino acid located in the second transmembrane domain. In GluA2, the residue is either glutamine (in the unedited form) yielding a receptor which conducts Ca^2+^ as well as monovalent cations, or arginine (in the edited form) which confers low Ca^2+^ permeability (Hume et al., 1991; Verdoorn et al., 1991). Co-assembly of edited GluA2 with GluA1, 3 or 4 subunits prevents Ca^2+^ entry through the receptor channel. In neurons, the efficiency of editing is high and thus the Ca^2+^ permeability of the majority of AMPA receptors is negligible; however, in malignant brain tumors, the editing efficiency is substantially reduced (Maas et al., 2001). Similarly in pediatric glioblastomas, only 50 to 70% of the GluA2 RNA is edited (Venkatesh et al., 2019), likely augmenting the Ca^2+^ signaling pathways that have been implicated in glioblastoma proliferation and migration.

## Voltage-Gated Ion Channels Enriched in Glioblastoma

Voltage-gated Ca^2+^ (Ca_V_) channels are involved in diverse functions, including regulating the entry of Ca^2+^ to control the release of neurotransmitters and hormones. Ca_V_ channels are composed of a number of subunits encoded by separate genes; the largest alpha-1 subunit forms the ion pore, and is associated with alpha-2, beta, gamma and delta auxiliary subunits that modulate functional properties. The Ca^2+^ channel-related genes showing transcript levels enriched in glioblastoma (**Table 1**) were Ca_V_ gamma3, -7, and -8. Ca_V_ alpha subunits were not represented in the GBM-enriched category, perhaps hinting at a role for gammas other than voltage-gated Ca^2+^ entry. As reviewed by Snutch and colleagues (2000-2013), there are eight types of Ca_V_ γ subunits. One of the first to be discovered was stargazin (classified as γ2), named after the Stargazer mouse strain which is prone to absence seizures caused by a mutation in the gamma2 gene. It is intriguing to note that gamma subunits γ3, γ7, and γ8 are expressed in brain and have been found to serve as transmembrane AMPA receptor regulatory proteins, known as TARPs (Tomita et al., 2003; Yamazaki et al., 2010). TARPs γ2 and γ7 cooperatively promote AMPA receptor expression in cerebellum, and γ7 also promotes expression in glia (Yamazaki et al., 2010). The primary AMPA receptor found in the hippocampus is a heteromeric combination of GluA1 and 2 subunits, associated with two γ8 auxiliary subunits which modulate gating by interacting with the GluA2 ligand-binding domain (Herguedas et al., 2019). The interaction of GluA2 receptors with TARP γ8 shifts the AMPA receptor channel conductance to a higher level (Carrillo et al., 2019). It will be interesting to test whether the augmentation of glutamate signal amplitudes by increasing expression or conductance is a key adaptive mechanism favoring gamma subunit expression in glioblastoma.

Based on transcriptomic data, the voltage-gated Na^+^ (Na_V_) alpha subunits 1, 2, and 3 appear to be expressed in glioblastoma at the RNA level. In excitable cells the primary role of Na_V_ channels is to generate action potentials that can be conducted over distances, harnessing a regenerative loop fueled by the Na^+^ gradient. The Na^+^-dependent action potential typically links to voltage-gated Ca^2+^ entry to generate biological work such as the release of neurotransmitters, secretion of hormones, or contraction of muscle, for example. The reported absence of measurable action potentials in identified GBM cells (Venkataramani et al., 2019) however invites speculation on alternative hypotheses, including possibilities that Na_V_ protein might not be assembled or functional, its distribution in plasma membrane might lack a requisite spatial organization, or shunting of currents through the gap junction-linked network might render depolarization responses undetectable. In the U87 GBM cell line, the effectiveness of a Na_V_ channel blocker found to reduce migration and invasiveness was not dependent on Na^+^ channel inhibition, not replicated by application of tetrodotoxin, and was suggested to be due to side effects on RNA demethylase (Malacrida et al., 2020). In general, Na_V_ channels serve as a mechanism for the amplification of small excitatory signals into large amplitude depolarizations, and in principle might contribute a role in boosting excitatory responses in glioblastoma, but a logical role for Na_V_ channels in GBM remains to be proposed for testing.

Voltage-gated K^+^ channels are a large diverse group of channels that serve to maintain the negative resting membrane potential in many different types of cells, and to restore the resting voltage during the repolarization phase of action potential responses in excitatory cells such as neurons, muscle and sensory cells. Ca^2+^ dependent K^+^ channels add the capacity to shape membrane potential by controlling the amplitude and kinetics of repolarizing K^+^ currents in response to both voltage and intracellular Ca^2+^ levels. K^+^ channels identified at moderate transcript levels (**Table 1**) include the voltage-gated Kv4.2 (KCND2), an inactivating A-type channel expressed in brain and heart, and Kv7.2 (KCNQ2), which alone or with KCNQ3 forms M channels in the central and peripheral nervous system. M-currents activate and deactivate slowly, regulating excitability in neurons. Channel closure by Gq-protein-coupled muscarinic acetylcholine receptors enables repetitive firing (Brown and Passmore, 2009). The Ca^2+^-activated KCa2.2 (KCNN2) and 2.3 (KCNN3), members of the small conductance class labeled SK, are more sensitive to Ca^2+^ and less sensitive to voltage than the big conductance BK channels, and contribute to hyperpolarized interludes in neurons following bursts of firing, as reviewed by Kaczmarek and colleagues (2017). In glioblastoma, a proposed purpose of cation efflux through K^+^ channels (in parallel with anion efflux though Cl^-^ channels) is to induce osmotic shrinking, decreasing glioma cell volume to expedite invasiveness through confined spaces (Olsen et al., 2003). Alternatively, in the absence of a substantial Cl^-^ leak, K^+^ currents would be expected to buffer membrane potential at a negative voltage, maintaining the driving force for Ca^2+^ entry (through pathways including for example, the Ca^2+^ permeable glutamate receptors). Relatively high transcript levels in glioblastoma were indicated for the inward rectifier Kir4.1 (KCNQ2), summarized in **Table 1**. This channel is expressed in brain and kidney. In brain oligodendrocytes, Kir4.1 channels are clustered near nodes in the myelin, where they are thought to remove accumulations of K^+^ near electrically active membranes (Schirmer et al., 2018). Loss of function of Kir4.1 is associated with axonal degeneration. The pattern of subcellular localization of the water channel AQP4 in glial cells coincides with that of the potassium channel Kir4.1, suggesting a coordination of activities for the spatial buffering of K^+^ and water homeostasis (Nagelhus et al., 2004; Yool, 2007).

## Synaptic Proteins Enriched in Glioblastoma

Since the synapses onto glioblastoma cells are made by neurons, and reverse synapses have not been observed (Venkataramani et al., 2019), at first glance it seems a mystery why glioblastoma cells would have measureable levels of transcripts for presynaptic proteins. However, a careful evaluation of the candidate genes indicates that these proteins might not be exclusively limited to presynaptic functions. Synaptic proteins in glioblastoma (**Table 1**) with moderate transcript levels include NSG2 and SYN1. Neuronal vesicle trafficking associated 2 (NSG2) protein is abundant in the dendrites and cell bodies of developing neurons, but its role until recently was unknown. Chander and colleagues discovered Nsg1 physically interacts with GluA1 and GluA2 in postsynaptic domains, where it promotes synapse formation, GluR expression, and synaptic activity (Chander et al., 2019). Synapsin I (SYN1) is regarded as a reliable presynaptic marker for immunocytochemistry. In axonal nerve terminals, it associates with synaptic vesicles to hold them in a reserve (non-released) pool. Phosphorylation in response to Ca^2+^ influx causes synapsin I to dissociate, freeing the vesicle for docking and transmitter release. Immunostaining and northern analyses have shown SYN1 protein and mRNA can expressed outside of neural tissues, in liver, epithelia, and cell lines, in which it is associated with the Golgi complex rather than plasma membrane, suggesting involvement in protein trafficking (Bustos et al., 2001). Synapsins have been identified in a variety of non-neuronal cells (Cesca et al., 2010) including chromaffin cells, PC12, immature astrocytes, pancreatic beta cells (in which synapsin I is associated with secretory granules), and in osteoblasts which are able to trigger glutamate release by processes mimicking neurotransmission (Bhangu et al., 2001).

It is interesting to note that human glioblastoma cells can release sufficient glutamate to raise extracellular concentrations up to 600 µM, which promotes GBM cell motility and growth, and causes excitotoxic cell death and seizures in peritumoral brain tissues (Corsi et al., 2019). The glutamate release process could be mediated by entirely by amino acid exchangers; but atypical neurotransmission-like mechanisms remain to be explored.

High transcript levels in glioblastoma (**Table 1**) were measured for SNAP-25, the synaptic vesicle glycoprotein 2A (SV2A), and for synaptophysin. Synaptosome associated protein 25 (SNAP-25) is involved in the control of synaptic vesicle exocytosis, and is essential for synaptic transmission (Jahn et al., 2003). SNAP-25-mediated effects do not point exclusively to a presynaptic role; it also has been suggested to govern postsynaptic morphology (Fossati et al., 2015) and receptor trafficking (Selak et al., 2009; Lau et al., 2010; Jurado et al., 2013) though some details remain controversial (Antonucci et al., 2016). SV2A, expressed throughout the brain, is a binding site for some anti-epileptic drugs such as levetiracetam, and is differentially associated with GABAergic and glutamatergic synapses, depending on the brain region, to regulate the balance of excitatory and inhibitory inputs (Venkatesan et al., 2012; Vanoye-Carlo and Gomez-Lira, 2019). Knock out of this gene in mice gives rise to violent spontaneous seizure activity leading to early death. Although viewed as a marker of neuronal synaptic vesicles, synaptophysin protein (SYP) was identified in medulloblastoma, neuroblastoma, and other tumors more than 30 years ago (Schwechheimer et al., 1987). It is linked to endo- and exocytotic mechanisms including formation of a fusion pore for the release of neurotransmitter, though genetic knock out does not result in an overt phenotype (McMahon et al., 1996). Continuing work has found synaptophysin immunoreactivity to be a diagnostic and prognostic tool in a wide array of cancer types, but the functional role of the protein remains unclear.

Very high transcript levels in glioblastoma (**Table 1**) were measured for synaptotagmin-11. Synaptotagmins generally are associated with evoked release of synaptic and secretory vesicles, as low affinity Ca^2+^ sensors that can bind to syntaxin and SNAP-25 (Burgoyne et al., 2019). Synaptotagmin 11 (Syt11), a substrate for ubiquitinylation by parkin, is one of the risk genes for Parkinson's disease; overexpression (resulting from parkin dysfunction) leads to impaired dopamine release, degeneration of dopaminergic neurons, and associated motor behavioral deficits (Wang et al., 2018a). These findings indicated a role for synaptotagmin-11 in the inhibition of endocytosis and vesicle recycling in neurons. Another member of the synaptotagmin family, Syt3 was shown to interact with GluA2 receptors in postsynaptic membranes, where it enhanced the endocytosis of receptors in response to Ca^2+^, generating an activity-dependent decrease in synaptic strength (Awasthi et al., 2019). It would be interesting to test the idea that Syt11 in glioblastoma slows endocytosis of GluA2 receptors, promoting glutamate-driven signaling.

## Transcriptomic Analysis of Additional Channels and Transporters Linked to Glioblastoma

**Figure 3:**
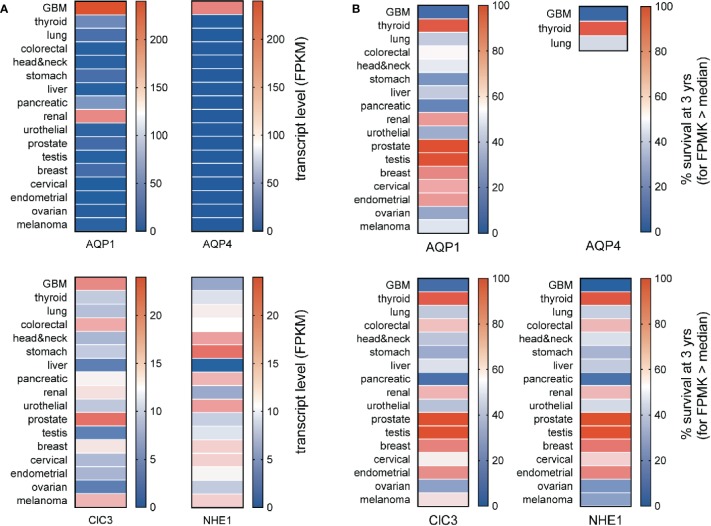
Comparison of transcript levels across different cancers. **(A)** Heat map comparison of median transcript levels (FPMK, color bars) across a panel of different cancers. **(B)** Heat map comparison of patient survival at 3 years (%, color bars) across a panel of different cancers. Results were calculated from TCGA data available in the Human Protein Atlas. TCGA, The Cancer Genome Atlas.

The specificity of enhanced channel and transporter gene expressions as markers for GBM can be assessed by comparing median transcript levels for a protein of interest across a broad panel of cancer types (**Figure 3A**) and assessing whether expression at levels greater than the median correlates with a poorer prospect of survival (**Figure 3B**). Aquaporins 1 and 4 (AQP1 and AQP4) are both strongly elevated in GBM; AQP1 is also upregulated in renal cancer. The high levels of AQP1 and AQP4 seen in GBM correlate with a poor chance of survival that is characteristic of the GBM diseases (**Figure 2B**). Conversely, the low levels of AQP1 and absence of detectable AQP4 transcripts in stomach and pancreatic cancers do not appear to offer any protective advantage in terms of survival, and the higher level of AQP1 in renal cancer does not coincide with a higher risk of death.

The expression of AQP1 in the leading edges of migrating cells has been linked to enhanced motility, as documented in neural crest (McLennan et al., 2020) and various types of cancer cells including colon cancer, gliomas, breast cancer, and endothelial cells involved in angiogenic responses (McCoy and Sontheimer, 2007; Pei et al., 2016a; Nakhjavani et al., 2019; Tomita et al., 2019). AQP1 is a dual water and ion channel (Anthony et al., 2000; Yu et al., 2006; Campbell et al., 2012; Kourghi et al., 2018), which has been suggested to accelerate cell migration by enabling parallel water and cation entry (Yool et al., 2009; Pei et al., 2019), promoting the cellular lamellipodial and filopodial extensions needed for forward movement. Inhibition of either the AQP1 water pores (in each subunit) or the ion channel (thought to reside in the central pore of the tetramer) slows migration in AQP1-expressing cancer cell lines (Kourghi et al., 2016; Pei et al., 2016b), and the inhibition of both shows an enhanced blocking effect on motility (De Ieso et al., 2019).

Transcript levels for the chloride channel ClC3 are seen across multiple classes of cancers, with the highest in prostate cancer, fairly high values in GBM, melanoma and colorectal cancers, and moderate levels in others. Molecular knockdown of ClC3 channels in glioma cells decreased Cl^-^ efflux, and slowed the volume decrease associated with DNA condensation in mitosis, suggesting a role for this conductance in promoting proliferation (Habela et al., 2008). Block of outwardly rectifying Cl^-^ currents in U251MG cells with chlorotoxin reduced motility in invasion assays *in vitro* and in rat cortical brain slice assays, suggesting glioma cell motility might be enhanced by a Cl^–^mediated decrease in cell volume, facilitating penetration through extracellular matrices (Soroceanu et al., 1999). Subsequent work showed the site of action of chlorotoxin in glioma invasion was *via* the selective inhibition of matrix metalloprotease-2 (Cao et al., 2013). Chlorotoxin binds preferentially to cancer cells including gliomas (Lyons et al., 2002), and its promise as a selective histopathological marker has proven valuable for targeting nanoparticles *in situ* in human GBM xenografted mice (Stephen et al., 2019), opening exciting possibilities for GBM cell-targeted delivery of chemotherapeutics (Fang et al., 2015).

The sodium proton exchanger NHE1, though a negative prognostic indicator for GBM, is most strongly expressed in cancer types such as head and neck, stomach, pancreatic, urothelial, and comparatively low in GBM. Nonetheless, increased levels of NHE1 expression found in the lamellipodial leading edges in primary glioma and GBM cell lines including U87, U251 and U118 are associated with increased export of protons, resulting in a more acidic environment and basic intracellular pH, associated with resistance to temozolomide, and enhanced tumor progression (Cong et al., 2014).

Other candidates meriting further analysis include gap junction channels (connexin 26) and bestrophin. Connexin 26, Cx30 and Cx43 are characteristic components of the majority of gap junctions between astrocytes in the rat central nervous system (Rash et al., 2001). Bestrophin Ca^2+^-activated anion channels are permeable to chloride as well as large organic anions such as glumate and GABA; they are expressed throughout the brain and in the retinal pigment epithelium of the eye, in which inherited mutations are associated with Best's type of macular degeneration (Oh and Lee, 2017). Roles of these channels in GBM remain to be determined.

## Prognostic Value in Evaluating Sets of Signaling Proteins

**Figure 4:**
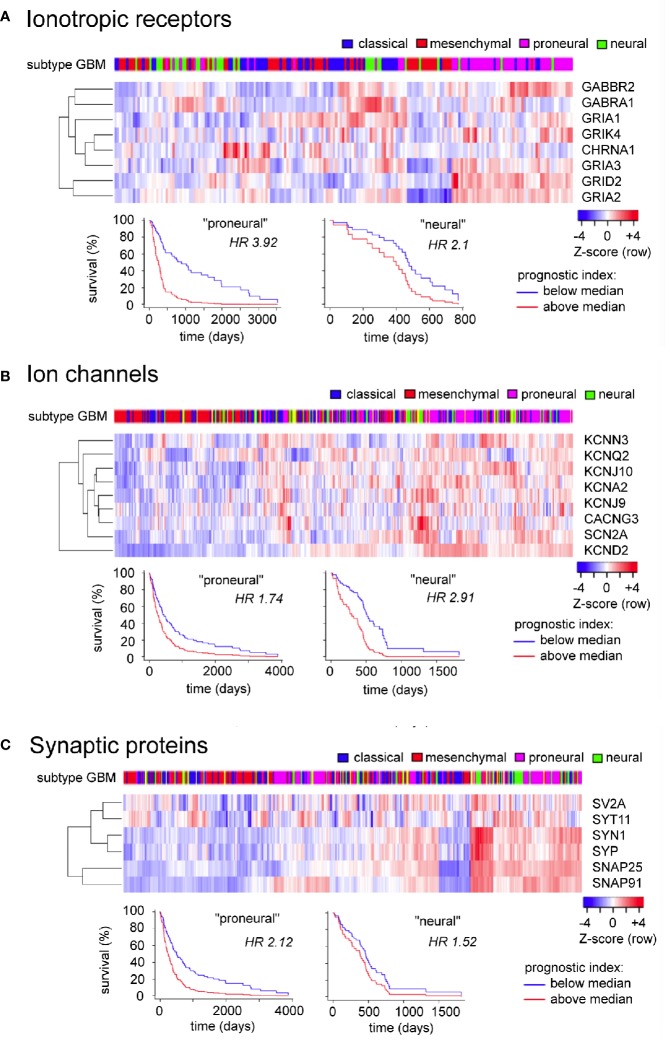
Evaluation of expression patterns and additional prognostic information available in analyzing sets of related signaling genes, for ionotropic neurotransmitter receptors **(A)**, ion channels **(B)**, and synaptic proteins **(C)** enriched in glioblastoma. Data were analyzed using the GBM Bio Discovery Portal, a resource for user-specified interrogation of The Cancer Genome Atlas (TCGA) database, for glioblastoma multiforme categorized by molecular subtype (Verhaak et al., 2010). GBM, glioblastoma multiforme.

The GBM Bio Discovery portal provides an interface for evaluating defined sets of multiple genes as prognostic indicators, drawing on the Human Protein Altas TCGA transcriptomics database. For the analysis here, clusters of genes were created from the GBM-enriched genes summarized in **Table 1** that had strong reliability scores (i.e., links to GBM based on multiple lines of evidence including immunocytochemistry, protein and RNA analyses, as denoted by “pathological evidence” scores of 1.0). The three clusters created were ionotropic neurotransmiter receptors, ion channels, and synaptic proteins (**Figure 4**). Data are presented as heatmaps (above), and as Kaplan Meier survival curves (below) for each cluster. Each column in the heat map represents data from a single GBM patient, and illustrates the different transcript levels measured for the genes in the cluster, presented as Z-scores relative to the average transcript level across the GBM sample population. Subtypes of glioblastoma assigned per patient are depicted by a color key (top row). Column order in the heat map columns reflects overall transcript levels, with predominantly low (blue) on the left side of the heat map, ranging to high (red) on the right side.

The subtype categories (neural, proneural, mesenchymal, and classical) serve as a useful tool for TCGA data analysis but do not reflect a consensus opinion; evidence for the neural classification has been questioned (Behnan et al., 2019) leaving support for three subtypes only. Limitations of the TCGA database include the fact that many of the samples analyzed were taken from single random sites in the tumor bulk, not capturing the inherent heterogeneity of subtypes across different regions within the tumor (Puchalski et al., 2018). However, biological relevance for the classification scheme has been supported by similarities in expression profiles of glioma cells with those of brain cell populations, prompting the idea that a common source for the gliomas might be pleuripotent neuronal stem cells of adult brain, which become glioblastoma stem cells and differentiate into subtypes based on environmental and genetic factors. Proneural cells show an oligodendrocyte progenitor cell-like signature, and classical and mesenchymal subtypes reflect astrocyte-like lineages (Verhaak and Valk, 2010; Alcantara Llaguno et al., 2015).

For the Ionotropic receptors (**Figure 4A**) and Ion channels (**Figure 4B**) clusters, the samples with predominantly high levels of expression (right side) correlated most frequently with GBM subtypes diagnosed as proneural in character. For the Synaptic proteins cluster (**Figure 4C**), the high expression patterns correlated with both proneural and neural subtypes of GBM. The branched tree (left side of the heat map) summarizes the degree of covariance in transcript levels for genes within the cluster. The survival plots in each cluster demonstrated that the prognostic impact of expression of a set of genes was stronger than that of any individual gene alone, as indicated by Hazard Ratio (HR) scores nearing as high as four for clusters (i.e., a 4-fold increase in chance of death in 3 years for transcript levels > median), as compared with hazard ratios close to 1.0 (ranging mainly 0.8 to 1.2) when evaluated for individual genes.

**Figure 5:**
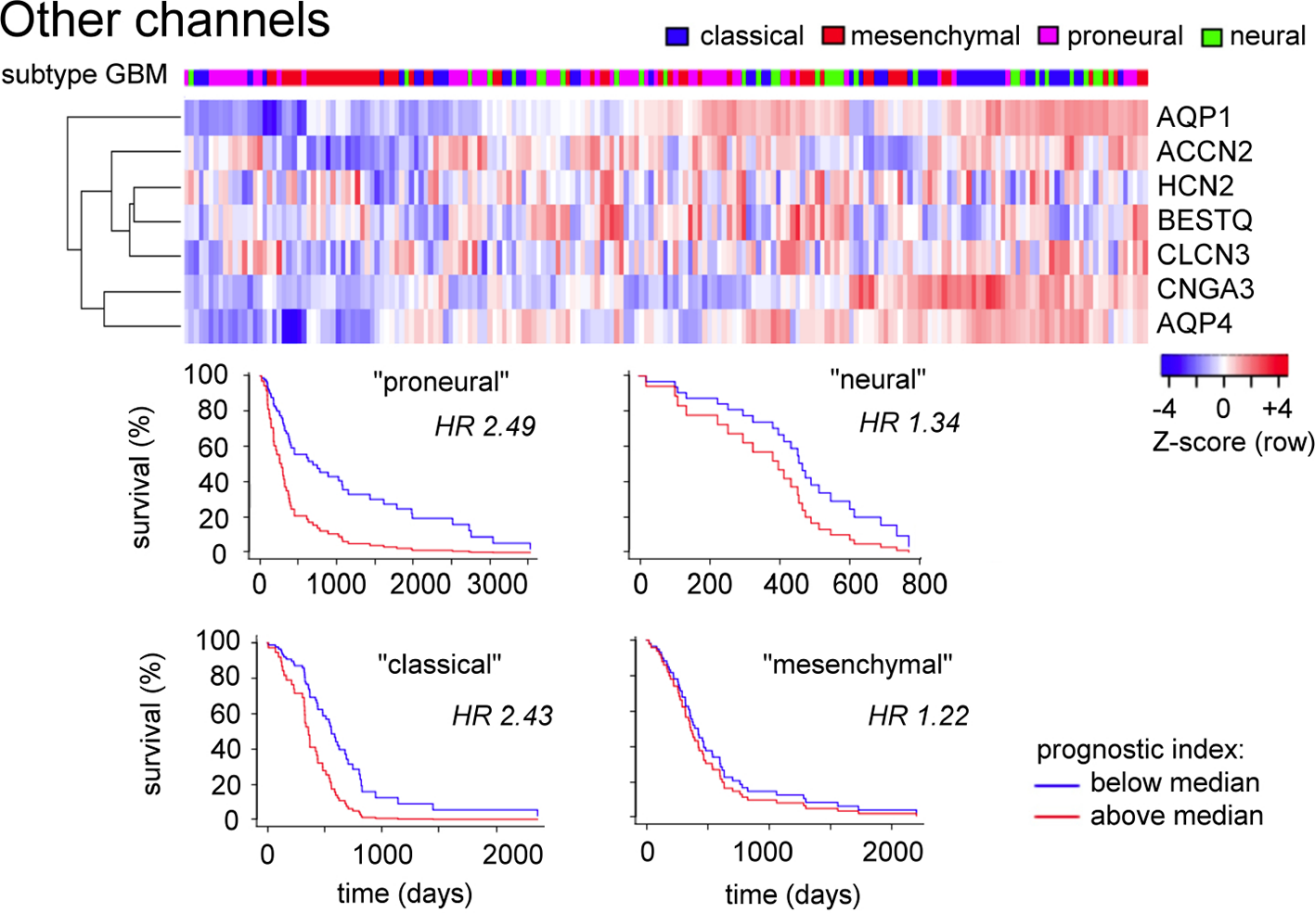
Evaluation of expression patterns for additional classes of channels enriched in glioblastoma. Data were analyzed using the GBM Bio Discovery Portal. GBM, glioblastoma multiforme.

A comparable analysis of genes in the ‘Other channels and transporters' cluster (**Figure 5**) based on **Table 1** (not limited to pathological evidence scores of 1.0) shows that patterns of high transcript levels in this group correlated most strongly with the classical and proneural subtypes of GBM, with hazard ratios of 2.4 to 2.5 determined from survival curves.

Transcriptomic data analyses indicated that the proneural subtype is consistently correlated with patterns of transcript levels across several clusters of membrane signaling proteins associated with GBM, and illustrate the point that calculations of hazard ratios for related groups of genes in GBM could have a stronger prognostic significance with respect to evaluating patient survival time than predictions based on expression levels of single genes alone. This insight at the genomic level is likely to be meaningful from a functional point of view as well. A major limitation that must be kept in mind in these analyses is that the tumor biopsy samples used for generating the RNAseq database were taken from random locations in the tumor, and thus are unlikely to capture the full heterogeneity and range of cell phenotypes within the tumor mass, complicating determination of correlations with patient survival and transcript prognostic value. Nonetheless, the transcriptomic analyses highlight the concept that cellular signaling is carried out by sets of proteins with partial overlap in functional properties, providing cells with capacity for compensation following the loss of a given signaling component. Therapeutic strategies aimed at modulating multiple proteins within a class, and multiple targets across classes, might prove to be more effective in realizing a clinically relevant outcome. A key point is that an ideal treatment, tailored to a given cancer, would incorporate multiple pharmacological agents targeted across a set of key proteins, each administered at low doses to reduce the severity of side effects, but achieving potency by a synergistic complementation. In this regard, it will be of interest to screen herbal medicinal extracts comprising naturally complex combinations of chemical components, for effects on a panel of different classes of signaling proteins for potentially synergistic interactions.

## Natural Plant Products as Sources of Therapeutic Agents

The current therapies for glioblastoma involving temozolomide chemotherapy, radiation, and surgical resection yield only limited improvements in survival periods. New drugs that induce fewer side effects and lead to more favorable prognoses are needed. Plant extracts have since ancient times been used traditionally as medicines in Egypt, China, India, Greece, and other countries. Some of the first written records on the medicinal uses of plants date back to 2600 BC. The plant kingdom comprises nearly 250,000 plant species of which only around 10% have been screened thus far for different diseases. Medicinal herbs found by experience to be valuable for treating diseases and ailments have served as original sources for many clinical compounds in the current therapeutic armamentarium. The National Cancer Institute (USA) has screened 35,000 plant samples (114,000 extracts) from 20 countries for anti-cancer activity (Developmental Therapeutics Program, NIH, USA). Approximately 60% of the clinically approved anti-cancer drugs available commercially are derived from medicinal plants (Khan, 2014). Many studies have considered compounds as effective potential anticancer treatments if they arrest the cell cycle or trigger apoptosis (reducing proliferation), impair angiogenesis (reducing metabolic supply), or decrease the expression of matrix metalloproteinases (impairing invasion) as reviewed by Park, Erices, and colleagues (Park et al., 2017; Erices et al., 2018). Ability to cross the blood brain barrier would facilitate drug delivery to the tumors.

Natural plant products tested in glioblastoma including flavonoids, terpenoids, polyphenols, epigallocatechin gallate, quinones, saponins, and others (**Table 2**), have been assayed mainly in glioblastoma cell lines, as well as xenografts in mice. A diverse set of plant products are effective in slowing proliferation and migration, and increasing cell death. The molecular mechanisms underpinning the effect of these compounds include decreased expression of angiogenic factors (VEGF and TGF-β1), disruption of signaling pathways (PI3K/AKT/mTOR, RAF-MEK-ERL, TLR4, IL-6 mediated JAK/STAT3, and Rho/ROCK), impaired mitosis (cell cycle arrest at G2/M phase), decreased expression of proliferation-associated genes (PCNA and cyclin D1), enhancement of apoptosis by increased Caspases (CASP 3, 9) and PARP 1 cleavage activities, decreased anti-apoptotic protein (Bcl-2) expression, downregulation of the activity of matrix metalloproteinases (MMP 2,9,14,16), and inhibition of actin assembly (see references in **Table 2**).

**Table 2:** Summary of alternative medicinal compounds and extracts reported to affect proliferation, viability, and/or motility of glioblastoma and malignant brain tumor cells.

Name	Plant Source	Compound	Chemical Structure	Targets	Treatments	Effects	Cell lines	References
Avishan-e-Shirazi	*Zataria multiflora* (thyme-like plant)	Thymol		Apoptosis; DNA fragmentation	Zataria extract (25–200 µg/ml) + ionizing radiation (3Gy and 6 Gy)	Inhibit cell proliferation; radiosensitize cells; increase apoptosis	A172	(Aghamohammadi et al., 2015)
Angelic root/female ginseng	*Angelica sinensis* (dong quai)	Phytosterols, polysaccharides, flavanoids		Apoptosis; proliferation; cell cycle	Chloroform extracts (IC50 = 3–47 µg/mL)	Induce apoptosis; arrest cell cycle at G0–G1; activate procaspase 9 & 3; decrease levels of phosphorylated Rb proteins; decrease tumor growth,	DBTRG-05MG, RG2 rat GBM cells, G5T/VGH GBM, GBM 8401, GBM8901 cells,	Tsai et al. (2005)
Berberine	*Berberis vulgaris* (barbary fruit); *Hydrastis* *canadensis* (goldenseal); *B. aquifolium* (Oregon grape); *Coptis chinensis* (goldthread); *Radix scutellariae* (skullcap)	Isoquinoline alkaloid	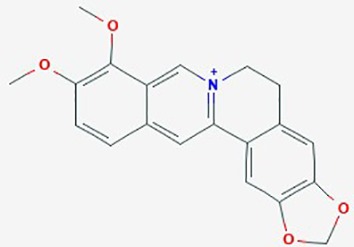	**1**. Cell-cycle; apoptosis **2**. EGFR-MEK-ERK signaling; proliferation,	**1**. Berberine (50 – 200 µg/ml) **2**. Berberine (0–150 µM), TMZ (0–320 µM)	**1**. Decrease cell viability (dose dependent); increase ROS & Ca^2+^; induce ER stress; in increase ratio Bax/Bcl-2 proteins; activate caspase-9 and -3 and cleavage of PARP **2**. reduce EGFR; inhibit RAF-MEK-ERL signaling; induce senescence; IC_50_ 6-fold lower than TMZ	U87, U251, U118, T98G	1.(Eom et al., 2008) 2. (Liu et al., 2015)
Betulinic Acid	*Betula alba* (White-barked birch)	Pentacyclic triterpenoid	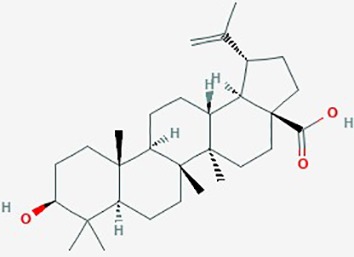	**1**. Apoptosis; BAX and Bcl-2 family of proteins; caspase-PARP cascade **2**. NF-Kb; apoptosis **3**. apoptosis **4**. apoptosis	**1**. Betulinic acid **(**0–250 µM ( **2**. Betulinic acid **(**0–10 µM) **3**. Betulinic acid (0–8 µg/ml) + TRAIL (0–10 ng/ml) **4**. Betulinic acid **(**1–100 µg/ml), effective concentrat'n *in vitro* & *in vivo* ranged 2–17 µg/ml	**1**. increase BAX and Bcl-2 proteins, formation of ROS; apoptosis, EC_50_ 20 µM) **2**. activate NF-kB (dose dependent); promote Bet A induced apoptosis, **3**. increase cleavage of caspase-8 and Bid by combined betulinic acid and TRAIL; increase apoptosis **4**. Bet A induced apoptosis (dose and time dependent); proteolytic degradation of caspase -8, -3 and PARP	LN229, U273,A172, U118MG, U138MG, U251MG, U343, U373, SK14, SK17, SK19, SK22, SK49, SK51, SK55, SK60	**1**. (Wick et al., 1999) **2**.(Kasperczyk et al., 2005); **3**. (Fulda et al., 2004) **4**. (Fulda et al., 1999)
Bittersweet	*Celastrus* *orbiculatus* (Oriental bittersweet)	Terpenoids	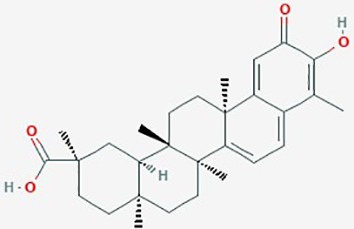	Cytoskeleton, N-cadherin, vimentin, MMP-2, MMP-9, E-cadherin genes;		**Inhibit migration and invasion** (dose dependent); reduce N-cadherin, vimentin, MMP-2 and MMP-9 expression; increase E-cadherin expression; inhibit actin assembly	U87; U251	(Gu et al., 2016)
Brazilin	*Caesalpinia sappan* (Sappan wood)	Red dye	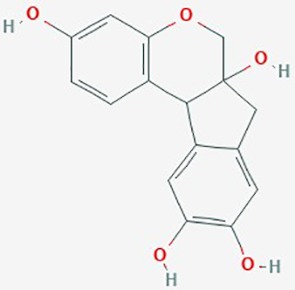	Cell cycle, caspase-3 and caspase-7; PARP	Brazilin (0–40 µM)	Decrease proliferation; apoptosis; cell cycle arrest at sub-G1 phase; decrease expression of caspase-3 and caspase-7; increase expression of PARP	U87	(Lee et al., 2013)
Cannabinoids	*Cannabis sativa*	11-nor-D9-Tetrahydro-cannabinol-9-carboxylic acid (THC)	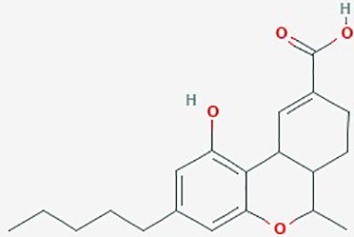	**1**. Cell cycle, apoptosis, ERK; Bcl family **2**. cell proliferation, apoptosis	**1**. THC (2 µM); **2**. THC (2.5– 3.3 µM) + Cannabidiol (0.6–1.2µM)	**1**. 9-THC inhibit proliferation; increase apoptosis **2**. induce ROS; down regulate phosphorylated ERK; combined THC and Cannabidiol synergistically inhibited cell proliferation	SG126, U87 MG, U251, SF188, U373 MG, U87	**1**. (Mc Allister et al., 2005); **2**. (Marcu et al., 2010)
Chokeberry	*Aronia melanocarpa*	Flavonoids/anthocyanins		Apoptosis, MMP-2, 14, 16 and 17	Extract (0– 600 µg/ml), effective at >200 µg/ml	Crosses BBB; induce necrosis; downregulate MMP-2, 14, 16 and 17 mRNA levels	U373	(Thani et al., 2012)
Crude extracts from ginger and Rhazya	*Zingiber officinale* (ginger); *Rhazya stricta* (evergreen shrub in Saudi Arabia)	Flavonoid/alkaloids		Apoptosis, Bax, Bcl-2, caspase-3 and -9, and PARP-1; NF-Kβ	Crude Rhazya extracts (0–200 µg/ml); Crude ginger extracts (0–200 µg/ml); or combined (0–50 µg/ml)	Suppress proliferation and colony formation; combined agents induce apoptosis; increase Bax : Bcl-2 ratio; enhance activities of caspase-3 and -9, and PARP-1 cleavage; downregulate NF-Kβ	U251	(Elkady et al., 2014)
Cucurbitacin B	*Trichosanthes kirilowii* Maximowicz (Chinese cucumber)	Terpene sterols	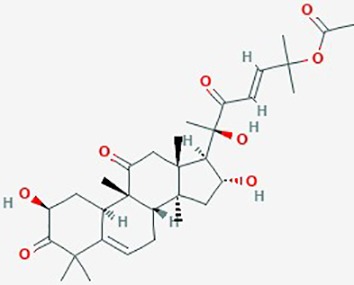	Cell cycle; JAK/STAT3 signaling pathways; cytoskeleton	10^-7^ M	Cell cycle arrest at G2/M phase; induce apoptosis; disrupt actin and microtubule network; **inhibit cell migration & invasion**	U87, T98G, U118, U343 and U373	(Yin et al., 2008)
Curcumin (Turmeric)	*Curcuma longa* (ginger family)	Diferuloyl-methane	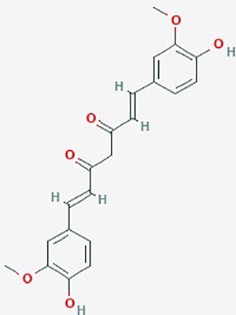	AKT/mTOR signaling; angiogenesis, MMP-9 expression	**1**. Curcumin (0–250 µg/ml), TMZ (0–250 µg/ml), or combined at (1.25 µg/ml) + (15.6 µg/ml) respectively. **2**. Curcumin (0–120 mg/Kg/day)	**1**. Combined treatment synergistically increased sensitivity to TMZ; enhanced apoptosis, increased ROS production, disrupted AKT/mTOR signaling pathways **2**. Crosses BBB;, decrease activity MMP-9, inhibit glioma angiogenesis; slow tumor growth; increase animal survival	**1**. U87MG xenografted mouse models **2**. U87MG xenografted into athymic mice	**1**. (Yin et al., 2014); **2**. (Perry et al., 2010)
Dioscin	*Dioscorea nipponica* Makino; *D. zingiberensis* Wright (yam family)	Saponin	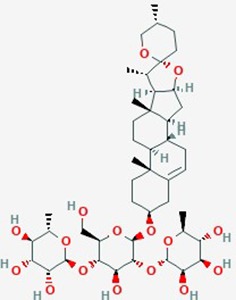	ROS, apoptosis, Bcl2, Bcl-xl genes, apoptosis	Dioscin (1.25 – 5 µg/ml)	Increase apoptosis (dose dependent); DNA damage; increase ROS; down regulate expression of Bcl2, Bcl-xl; inhibit proliferation	C6 gloma cells	(Lv et al., 2013)
Epigallocate-chin gallate	*Camellia sinensis* (tea flower)	Polyphenol	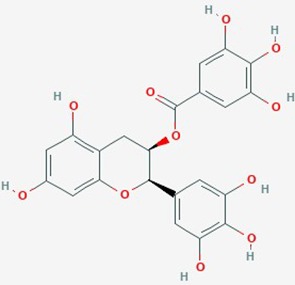	1. Matrix metalloproteinases, MMP2; 2. cell viability; apoptosis	**1**. EGCG (25 µM) **2**. EGCG (12.5, 25, 50 µg/mL)	**1**. Inhibit MMP-2 activation; reduce cell viability **2**. induce apoptosis; reduce viability	**1**. U87 MG; **2**. U373 MG; C6 glioma	**1**. (Annabi et al., 2002); **2**. (Yokoyama et al., 2001)
Ginseng	*Panax ginseng*	Ginsenoside Rg3	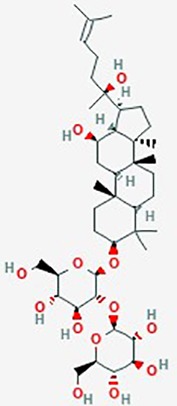	VEGF; Bcl-2; apoptosis	Rg3 (0–180 µg/ml), TMZ (0–180 µg/ml) Or Rg3 (10, 80 or -180 µg/ml) + TMZ (10, 80 or 180 µg/ml)	Downregulate VEGF and Bcl-2, suppress angiogenesis, combined TMZ with ginseng showed additive inhibition of proliferation (time and dose dependent); cell cycle arrest; increased apoptosis; reduced angiogenesis	Rat C6 glioma	(Sun et al., 2016a)
Honokiol	*Magnolia officinalis* (houpu magnolia)	Hydroxylated biphenol	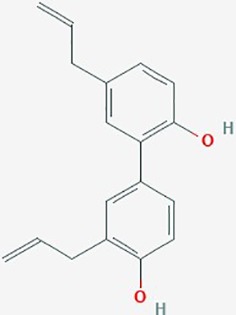	**1**. STAT3 signaling; ERK1/2, Bcl-Xl, autophagy **2**. proliferation, angiogenesis 3. Notch signaling, MGMT	**1**. Honokiol (6.25–50 µM) **2**. Honokiol (0–35 µg/ml) **3**. Honokiol (0–5 µM); TMZ (0–500 µM); or combined (10 µM) + TMZ (50µM)	**1**. Crosses BBB; increase apoptosis (dose dependent); decrease expression of Bcl-x; increase autophagy **2**. inhibit proliferation; increase survival(dose dependent); decrease angiogenesis **3**. inhibit proliferation (dose dependent); combined treatment enhanced apoptosis in GBM8401 SP cells; downregulate Notch3 signaling	**1**. DBTRG- 05MG **2**. U251, rat 9L gliosarcoma xenograft model **3**. GBM8401, U87 MG	**1**. (Chang et al., 2013); **2**. (Wang et al., 2011) **3**. (Lai et al., 2015)
Icariin (Horny Goat Weed)	*Epimediium species* dried leaf (yin yang huo, or horny goat weed)	Prenylated flavonol glycoside	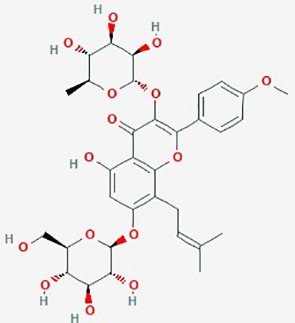	NF-κB activity	Icariin (10 µM) + TMZ (200 µM)	inhibit proliferation and apoptosis (dose-dependent); potentiate anti- tumor activity of TMZ; suppress NF-κB activity	U87MG	(Yang et al., 2015)
Iridin	*Iris versicolor*	Glycosyloxyi-soflavone	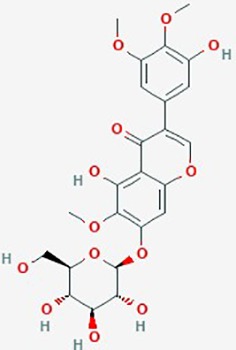	Glioblastoma-endothelial interactions	5 µM	inhibit intracranial tumor growth; increase median survival in xenograft mice; disrupt glioblastoma-endothelial interactions	U87	(Sengupta et al., 2015)
Kalamegha or Kalmega	*Andrographis paniculata* (AP)	Bicyclic diterpenoid	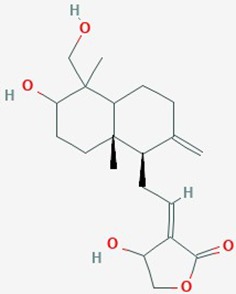	Cell cycle; Cdk1 and Cdc25C genes; PI3K/AKT/mTOR signaling pathway	10–100 µM	inhibit proliferation; inhibit PI3K/AKT/mTOR signaling pathway; decrease expression of Cdk1 and Cdc25C; G2/M cell cycle arrest	U87; U251	(Li et al., 2012)
OLE	*Olea europaea* (leaf extract)	Phenolic compound Oleorupin		**1**. miRNA **2**. angiogenesis MMP-2 and MMP-9 **3**. MGMT methylation, p53 expression	**1**.OLE (.005 –2 mg/ml), TMZ (325 or 450 µM) or OLE (1mg/ml) + TMZ (325 µM) **2**. OLE (2 mg/ml), bevacizumab (2.5 mg/ml) or combined OLE (2 mg/ml) + bevacizumab (2.5 mg/ml); **3**. OLE (1 mg/ml) or in combination with TMZ (350 µM)	**1**. Upregulate miRNA-181b, 153, 145, 137 & let-7d expression, increase apoptosis (i2 mg/ml); reduce angiogenesis; increase efficacy of TMZ when combined with OLE **2**. reduce expression of VEGF, MMP-2 and 9; reduce tumor weight; **reduce invasion and migration**; combined treatment synergistically enhanced effect of bevacizumab **3**. OLE (1 mg/ml) induced CpG island methylation in MGMT gene, combined with TMZ significantly increased toxicity on MGMT unmethylated cells	**1**. T98G **2** T98G and GSC+ cells from 5 GBM patients. **3**. Tumor cells from 21 GBM patients	**1**. (Tunca et al., 2012); **2**. (Tezcan et al., 2017a) **3**. (Tezcan et al., 2017b)
Osthole	Ripe cnidium fruits belonging to Umbelliferae	7-methoxy-8-isoamyl alkenyl coumarin	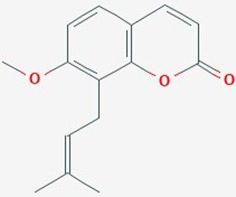	miR-16, MMP-9 signaling pathway, proliferation, apoptosis	Osthole (0–200 µM)	Upregulate miR-16; reduce protein expression levels of MMP-9; suppres proliferation; accelerate apoptosis	U87	(Lin et al., 2015)
Paeoniflorin	*Radix Paeoniae Alba* (*Paeonia lactiflora*)	Monoterpene glucoside	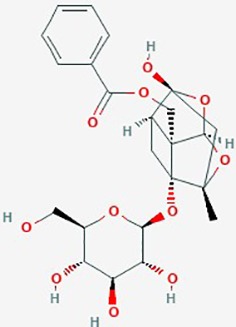	**1**. TLR4, TLAR4/Triad3 A pathway, ubiquitination proteasome pathway (UPP) **2**. HGF/c-Met signal, RhoA/ROCK signaling, cytoskeleton **3**. apoptosis, STAT3 signaling	**1**. Paeoniflorin (0–40 µM) **2**. Paeoniflorin (0–40 µM) **3**. Paeoniflorin (0–20 mM) **4**. Paeoniflorin (0–20 µM)	**1**. inhibit cell proliferation (dose dependent); downregulate TLR4; promote TLR4 degradation **2**. **inhibit HGF induced migration & invasion**, actin cytoskeleton organization *via* c-Met Rho/ROCK signaling; downregulate Rho **3**.apoptosis; inhibit proliferation (dose & time dependent); increase miR-16; reduce MMP-9; proteosome degradation of STAT3 and downstream Bcl-2 & *Survivin* **4**. accelerate apoptosis; reduce cell proliferation, increase miR-16, downregulate MMP-9	**1**. U87, U251, U118, T98G and U87 xenograft mouse model **2**. T98G; HA1800, HEB **3**. U87, U251 **4**. U87	**1**. (Wang et al., 2018b) **2**. (Yu et al., 2019) **3**. (Nie et al., 2015)
Plumbagin	Roots of Droseraceae, Plumbaginaceae & Ebenceae	Napthoquinone	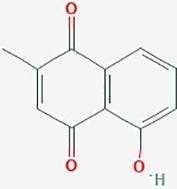	Cell cycle, apoptosis	Plumbagin (0–6 µM), KNS60 showed the highest sensitivity at a concentration of 3 µM	DNA damage; cell cycle arrest; apoptosis. Upregulate *PTEN, TNFRSF1A;* downregulate *E2F1*; lower MDM2, cyclin B1, survivin and BCL2 protein expression; inhibit telomerase; telomere shortening following chronic treatment	A172; U251; KNS60;	(Khaw et al., 2015)
Quercetin	Broccoli, red onions, apples, red grapes, cherries and berries	Flavonoid	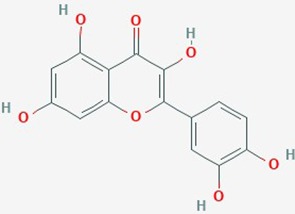	**1**. IL-6, STAT3 signaling, proliferation, apoptosis **2**. Hsp27, apoptosis **3**. cell cycle, apoptosis	**1**. Quercetin (0–50 µM) **2**. TMZ (200 & 400 µmol/L) alone or in combination with Quercetin (30 µmol/L); **3**. Quercetin (200 µmol/L)	**1**.Inhibit IL-6 mediated JAK/STAT3 signaling pathway(dose dependent); reduce proliferation and migration **2**.increase sensitivity of GBM lines to TMZ by suppression of heat shock protein 27 (Hsp27) **3**. increase caspase-3 & -7 proteolytic activity; induce autophagy (dose dependent); apoptosis	**1**. T98G; **2**. U87; U251, **3**. U373MG	**1**. (Michaud-Levesque et al., 2012); **2**. (Sang et al., 2014); **3**. (Kim et al., 2013)
Resveratrol	red wine Vitis, mulberry, peanuts	Polyphenolic phytoalexin	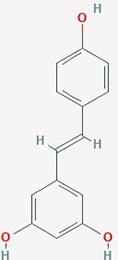	1. Notch 1 signaling, AKT; Bax2 expression G2/M cell cycle **2**. cell cycle, MMP-9 **3**. invasion **4**. extracellular matrix remodeling proteins, invasion **5**. apoptosis, NF-kB **6**. MGMT expression, apoptosis **7**. apoptosis	**1**. Resv (50 or 100 µM) **2**. Resv (10 µM), TMZ (100 µM); Resv (10 µM) + TMZ (100 µM) **3**. Resv (5–20 µM) **4**. Resv (1 or 50 µM) **5**. Resv (5 µM) **6**. Resv (100 µM), TMZ (100 µM) or combined **7**. Resv (20 or 40 µM), TMZ (100–1200 µM) or combined Resv (20 or 40 µM) + TMZ (200 or 400 µM)	**1**. Activate Notch-1 expression; induce AKT dephosphorylation; increase Bax and decrease Bcl-2 **2**.inhibit growth (dose dependent); reverse TMZ resistance by reducing MGMT; increase apoptosis; increase caspase-3 cleavage; G2/M cell cycle arrest; downregulate MMP-9; increase ROS; inhibit mTOR signaling; downregulate Bcl-2 **3**.**reduce TNF-induced invasion**; suppress NF-κB activity; downregulate uPA and its receptor **4**. decrease MMP-2 expression (dose dependent); decrease SPARC expression **5**. block activation of NF-kB (dose and time dependent); **6**. reverse TMZ resistance, increase apoptosis, decrease IC_50_ of TMZ, decrease NF-kB content **7**.enhance TMZ induced apoptosis	**1**. A172, T98G **2**. SHG44, *in vivo* rat model **3**. U373MG **4**. T60, T63 **5**. H4 **6**. T98G **7**. GICs from 2 patients	**1**. (Lin et al., 2011); **2**. (Yuan et al., 2012); **3**. (Ryu et al., 2011) **4**. (Gagliano et al., 2005); **5**. (Manna et al., 2000) **6**. (Huang et al., 2012) **7**. (Li et al., 2016a);
Rutin (faveira)	*Dimorphandra mollis*	Flavonoid	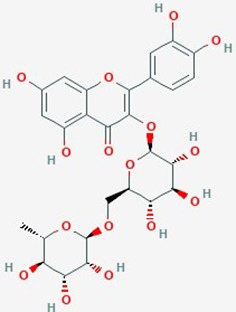	VEGF and TGF-β1	50 and 100 µM	Reduce levels of VEGF and TGF-β1 (reversed after 72h)	GL-15	(Freitas et al., 2011)
Silvery wormwood	*Artemisia argyi* (Chinese mugwort)	Trihydroxy-flavone		Cell cycle; apoptosis; Bax and p53 genes	30–300 µM	inhibit proliferation; promote apoptosis; cell cycle arrest in G2/M phase; increase pro-apoptotic protein Bax and p53 expression	U87	(Khan et al., 2012)
Tagitinin C Mexican sunflower	*Tithonia diversifolia*	Sesquiterpenoid	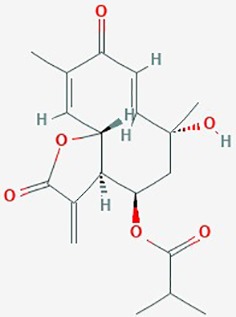	Cell cycle, *survivin*	0–10 µg/ml	inhibit viability *in vitro*; autophagic cell death and G2/M arrest	U373	(Liao et al., 2011)
Tetrandrine (Tet)	*Stephania tetrandra*	Bisbenzyliso-quinoline alkaloid	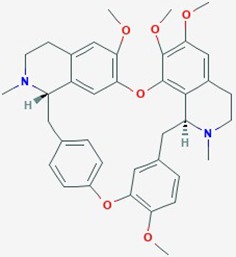	Cell cycle; ERK signaling pathway; PCNA and cyclin D1 genes	Tetrandirine (20 umol/L) + radiation (1.21 Gy/min)	inhibit cell proliferation; G0/G1 cell cycle arrest; attenuate radiation-induced ERK signaling; decrease expression of proliferation associated genes PCNA and cyclin D1	U87; U251	(Ma et al., 2017)
TQ	*Nigella sativa*	Thymoquinone		Autophagy, apoptosis	TQ (0–35 µM)	inhibit autophagy; induce cathepsin-mediated cell death	T98G; U87MG	(Racoma et al., 2013)
Withaferin A; Ashwagandha	*Withania somnifera*	Steroidal lactone	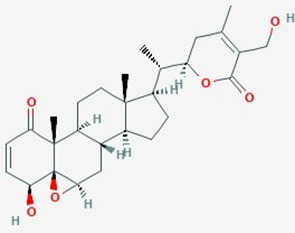	**1**. MGMT expression; Akt/mTOR pathway; cell cycle **2**. cell proliferation and migration **3**. apoptosis, cell cycle, cyclin D1, bcl-xl, p-Akt genes, NF-Kb, VEGF, HSP 70	**1**. Withaferin A (0.5–2 µM) alone or with TMZ (10–50 µM for sensitive, 100–500 µM for resistant lines) **2**. Withaferin **(**0.1–5 µM) **3**. Withania water extract	**1**. decrease proliferation; G2/M cell cycle arrest; deplete MGMT; reversed MGMT mediated TMZ-resistance; apoptosis *via* inhibition of Akt/mTOR pathway; cell cycle arrest **2**. inhibit proliferation; **delay migration** **3**. G2/M cell cycle arrest, increase apoptosis; suppress cyclin D1, bcl-xl, p-Akt genes, suppress NF-Kb, VEGF, HSP 70; reduced intracranial tumor volume	**1**. U87, U251, T98G, U87 TMZ, U251 TMZ **2**. C6 rat glioma, YKG1 **3**. Rat model of orthotopic glioma allograft	**1**. (Grogan et al., 2014) **2**. (Shah et al., 2009) **3**. (Kataria et al., 2016)
Xanthohumol	Hop cones of *Humulus lupulus L*.	Prenylated phenolic constituents	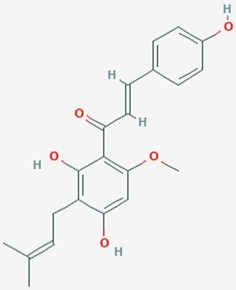	Apoptosis Caspase-3, 9 and PARP cleavage; Bcl-2 protein	20 µM	increase ROS; apoptosis activated by MAPK; down regulate Bcl-2 protein	T98G, U87-MG	(Festa et al., 2011)
γ-Mangostin	*Garcinia* *mangostana*	Xanthone	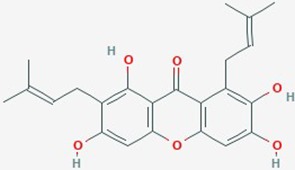	Cell cycle; apoptosis	10–200 µM	inhibited proliferation; increase hypodiploid cells; reduce viability (dose dependent); induce apoptosis; increase ROS production and mitochondrial dysfunction	U87; GBM 8401	(Chang et al., 2010)

Yellow highlighted boxes denote compounds with published effects on glioma migration and/or invasion. Chemical structures were downloaded from NINLB PubChem at https://pubchem.ncbi.nlm.nih.gov/

The limited number of natural plant compounds identified as inhibitors of motility in glioblastoma (**Table 2**) included bittersweet (*Celastrus orbiculatus*), curcubitacin B (*Trichosanthes kirilowii*), OLE (*Olea europaea* leaf), paeoniflorin *(Radix paeoniase alba)*, resveratrol (red wine and other sources), and withaferin A (*Withania somnifera*); however, it is important to note that potential anti-motility effects of other glioma-active plant preparations in **Table 2** are not excluded, but remain to be evaluated. A useful starting point is to survey whether any of the motility-blocking herbal compounds have published effects on ion channels or ionotropic receptors. Evidence is limited in this under-researched field, but available publications suggest possible links. For example, celangulin I isolated from bittersweet activated high-voltage activated L-type Ca^2+^ channels in insect neurons, consistent with excitoxicity and insecticidal activity (Li et al., 2016b). Cucurbitacin IIa decreased the expression of glutamate receptors GluN2B (NMDA) and GluA1 (AMPA), and increased GABA_A_α2 expression in mouse brain amygdala (Zhou et al., 2017). OLE from *Olea* inhibited L-type Ca^2+^ currents in rat cardiac cells (Scheffler et al., 2008), and reduced L-type Ca^2+^ channel RNA levels in rat spinal cord (Zare et al., 2012). Oleuropein from *Olea* caused dose-dependent increases in intracellular Ca^2+^ in mesothelioma cells, suggested to be mediated by activation of low-threshold T-type Ca^2+^ channels (Marchetti et al., 2015). An analgesic action of paeoniflorin was suggested to be mediated by activating adenosine A1 receptors and reducing glutamate release in rat pain sensory fibers (Zhang et al., 2009). Resveratrol has been found alter activity in an array of ion channel classes, including the inhibition of Ca^2+^ channels involved in pain perception pathways in mice, consistent with an anti-nociceptive effect of systemic treatment (Pan et al., 2017), inhibition of cardiac L-type Ca^2+^ currents in rat ventricular myocytes (Zhang et al., 2006), and activation of T-type Ca^2+^ channels in a mesothelioma cell line (Marchetti et al., 2016), providing an interesting parallel with the effects of *Olea* extracts listed above. Resveratrol activated defined subtypes of GABA_A_ receptors containing α1 subunits, expressed in *Xenopus* oocytes (Groundwater et al., 2015), and blocked the calcium-dependent K^+^ channel, KCa3.1, heterologously expressed in 3T3 fibroblasts (Olivan-Viguera et al., 2013). *Withania* extract activated mammalian brain GABA_A_ and GABArho receptors recorded by voltage clamp in the *Xenopus* expression system, evoking currents comparable to those induced by the native neurotransmittter; however, responses were not activated by the isolated components withaferin A and withanolide A, suggesting other active agents are present in the extract (Candelario et al., 2015). These observations point to an important gap in knowledge regarding the ion channel and receptor modulatory effects of anti-cancer medicinal herb extracts.

It is interesting to note that some compounds such as curcumin [1.25 µg/ml] (Yin et al., 2014), OLE extract [1 mg/ml] (Tezcan et al., 2017a), resveratrol [10 µM] (Yuan et al., 2012) and quercetin [30 µmol/L] (Sang et al., 2014) when tested in combination with temozolomide have been effective in increasing survival in animal models, overcoming temozolomide resistance by increasing ROS production, disrupting AKT/mTOR signaling, reducing MGMT gene expression, suppressing Hsp27 protein expression, and downregulating the expression of MMP-2 and -9. The plant derived compounds are effective in low doses and intriguingly the effective concentration of temozolomide required in combination is also reduced (50–100 µM) leading to more effective treatments with fewer side effects. Thus, natural products from traditional medicinal plants could prove useful in combination with other plant products or chemotherapy drugs, as a new way forward in treating glioblastoma tumors and improving prognoses, alone and in combination with front-line chemotherapeutic agents.

## Combining Modulators for Multiple Targets to Increase Selectivity and Potency of Treatments for Glioblastoma

Selected sets of membrane signaling proteins that are upregulated in glioblastoma might be added to the matrix of markers supporting diagnosis and targeted intervention. Moving from bench to bedside is challenging; many failures have resulted from approaches focused on single targets and single agents as therapies. Combinations of agents offer potential to focus treatments more precisely on a class of cells of interest, based on unique signature profiles reflecting expression of multiple key proteins from more than one essential pathway. However even combinations of approaches have not yet achieved the desired endpoints.

The standard current therapy for GBM does use a combined approach, employing radiotherapy, temozolomide chemotherapy and surgical resection. Studies have explored possible benefits of layering additional agents onto this standard therapy regime with the aim of further extending survival. Some logical pharmacological candidates such as gefitinib (an inhibitor of the EGF receptor) or bevacizumab (an inhibitor of angiogenesis) added to the standard therapy have not met the aims for improved overall survival (Okonogi et al., 2015).

With a diverse types of GBM-enriched proteins all appearing to point to a major role for glutamate signaling in glioblastoma, glutamate receptors are a clear target of interest for potential therapies, but have proven elusive thus far. Glutamate driven Ca^2+^ entry is linked to the invasion and growth, while release of excitotoxic glutamate in the local environment causes neuronal loss and further recruits tumor-associated microglia which produce growth factors enhancing tumor cell survival (Corsi et al., 2019). AMPA receptors are associated with integrin, and could contribute localized signaling at cytoskeletal anchor points in focal adhesion complexes (Piao et al., 2009) which might be key in invasive motility. A non-competitive antagonist of AMPA receptors, talampanel (also known as GYKI 53773 or LY300164) was found to increase the median survival of patients with GBM (20.3 months versus 14.6 months) in a cohort study of 60 patients (Grossman et al., 2009). Despite promise in Phase II, it has not been validated further and might be limited by a short half-life of 3 h (Langan et al., 2003). This agent, a derivative of benzodiazepine, also blocks kainate receptors (Donevan et al., 1994).

Targeting the AMPA glutamate receptors would seem to be a compelling approach. However, the predominant role of AMPA receptors in fast excitatory signaling throughout the central nervous system raises a challenge on how to target inhibitors selectively to cancer cells while maintaining tolerability. Most AMPA receptor antagonists tested thus far have proven unsuitable due to side effects and pharmacokinetics, in contrast to effectiveness in preclinical animal models (Lee et al., 2015). The natural extracts in traditional medicines that have been found to be effective might serendipitously include agents which modulate more than one of the classes of signaling molecules highlighted in this review, that are enriched in glioblastoma and could be important contributors to the pathological process. New approaches will be likely to exploit evidence-based combinations of selected agents, each at low doses, along with tools for cancer-selective targeting to GBM cells, to engineer new and potent therapeutic strategies for meeting the medical challenge of treating glioblastoma.

## Data Availability Statement

The datasets analyzed for this study can be found in the following repositories:

The Human Protein Atlas available from http://www.proteinatlas.org

The National Cancer Institute Glioblastoma Bio Discovery Portal available from https://gbm-biodp.nci.nih.gov/

The National Institutes of Health NCBI PubMed search platform available from https://www.ncbi.nlm.nih.gov/pubmed

The US National Library of Medicine PubChem available at https://pubchem.ncbi.nlm.nih.gov/.

## Author Contributions

All authors listed have made substantial, direct, and intellectual contribution to the work and approved it for publication.

## Funding

Support was provided by Australian Research Council Discovery Project grant 19ARC_DP190101745.

## Conflict of Interest

The authors declare that the research was conducted in the absence of any commercial or financial relationships that could be construed as a potential conflict of interest.
